# Acidification in the U.S. Southeast: Causes, Potential Consequences and the Role of the Southeast Ocean and Coastal Acidification Network

**DOI:** 10.3389/fmars.2020.00548

**Published:** 2020-07-10

**Authors:** Emily R. Hall, Leslie Wickes, Louis E. Burnett, Geoffrey I. Scott, Debra Hernandez, Kimberly K. Yates, Leticia Barbero, Janet J. Reimer, Mohammed Baalousha, Jennifer Mintz, Wei-Jun Cai, J. Kevin Craig, M. Richard DeVoe, William S. Fisher, Terri K. Hathaway, Elizabeth B. Jewett, Zackary Johnson, Paula Keener, Rua S. Mordecai, Scott Noakes, Charlie Phillips, Paul A. Sandifer, Astrid Schnetzer, Jay Styron

**Affiliations:** 1 Mote Marine Laboratory, Sarasota, FL, United States,; 2 Thrive Blue Consulting, Charleston, SC, United States,; 3 Grice Marine Laboratory, College of Charleston, Charleston, SC, United States,; 4 Arnold School of Public Health, University of South Carolina, Columbia, SC, United States,; 5 Southeastern Coastal Ocean Observing and Research Regional Association, Charleston, SC, United States,; 6 US Geological Survey, St. Petersburg, FL, United States,; 7 National Oceanic and Atmospheric Administration, Atlantic Oceanographic and Meteorological Laboratory, Miami, FL, United States,; 8 College of Earth, Ocean and Environment, University of Delaware, Newark, DE, United States,; 9 National Oceanic and Atmospheric Administration, Ocean Acidification Program, Silver Spring, MD, United States,; 10 National Oceanic and Atmospheric Administration, National Marine Fisheries Service, Southeast Fisheries Science Center, Beaufort, NC, United States,; 11 South Carolina Sea Grant Consortium, Charleston, SC, United States,; 12 United States Environmental Protection Agency, Gulf Ecology Laboratory, Gulf Breeze, FL, United States,; 13 North Carolina Sea Grant, Manteo, NC, United States,; 14 Nicholas School of the Environment and Biology Department, Duke University, Beaufort, NC, United States,; 15 Global Ocean Visions, LLC, Charleston, SC, United States,; 16 US Fish & Wildlife Service, Raleigh, NC, United States,; 17 Center for Applied Isotope Studies, The University of Georgia, Athens, GA, United States,; 18 Phillips Seafood, Sapelo Sea Farms, South Atlantic Fisheries Management Council, Townsend, GA, United States,; 19 Hollings Marine Laboratory, College of Charleston, Charleston, SC, United States,; 20 Marine, Earth and Atmospheric Sciences, North Carolina State University, Raleigh, NC, United States,; 21 Carolina Mariculture Company, Cedar Island, NC, United States

**Keywords:** coastal acidification, capacity-building, stakeholders, hypoxia, shellfish, crustaceans, coral

## Abstract

Coastal acidification in southeastern U.S. estuaries and coastal waters is influenced by biological activity, run-off from the land, and increasing carbon dioxide in the atmosphere. Acidification can negatively impact coastal resources such as shellfish, finfish, and coral reefs, and the communities that rely on them. Organismal responses for species located in the U.S. Southeast document large negative impacts of acidification, especially in larval stages. For example, the toxicity of pesticides increases under acidified conditions and the combination of acidification and low oxygen has profoundly negative influences on genes regulating oxygen consumption. In corals, the rate of calcification decreases with acidification and processes such as wound recovery, reproduction, and recruitment are negatively impacted. Minimizing the changes in global ocean chemistry will ultimately depend on the reduction of carbon dioxide emissions, but adaptation to these changes and mitigation of the local stressors that exacerbate global acidification can be addressed locally. The evolution of our knowledge of acidification, from basic understanding of the problem to the emergence of applied research and monitoring, has been facilitated by the development of regional Coastal Acidification Networks (CANs) across the United States. This synthesis is a product of the Southeast Coastal and Ocean Acidification Network (SOCAN). SOCAN was established to better understand acidification in the coastal waters of the U.S. Southeast and to foster communication among scientists, resource managers, businesses, and governments in the region. Here we review acidification issues in the U.S. Southeast, including the regional mechanisms of acidification and their potential impacts on biological resources and coastal communities. We recommend research and monitoring priorities and discuss the role SOCAN has in advancing acidification research and mitigation of and adaptation to these changes.

## INTRODUCTION

Ocean acidification generally refers to the oceanic pH decrease and associated changes in chemical speciation in dissolved inorganic carbon (DIC) that occurs as a result of absorption of atmospheric carbon dioxide. The ocean serves as a giant reservoir for CO_2_ and has absorbed about 31% of the CO_2_ emissions produced by humans (Sabine et al., 2004; Gruber et al., 2019), resulting in a significant increase in ocean acidity. It has been estimated that the acidity in surface ocean waters has increased by about 26% since 1860 resulting in a pH decline from 8.2 to 8.1 (Tanhua et al., 2015). Because the pH scale is logarithmic, a one-unit decrease in pH means a 10-fold increase in acidity. As atmospheric CO_2_ levels continue to rise, so will the amount of CO_2_ absorbed by the ocean, resulting in further acidification (Gattuso et al., 2015).

While atmospheric inputs are one contributing factor to acidification, coastal waters can have much higher CO_2_ concentrations than the open ocean; the atmosphere is not the only carbon source associated with changes in carbonate chemistry (Andersson and Mackenzie, 2012; Wallace et al., 2014). The carbonate system in coastal ecosystems is highly influenced by biological activity, freshwater inputs and organic carbon run-off from land. Furthermore, large storm events have been shown to have significant and long-lasting impacts on local and regional carbonate chemistry (Johnson et al., 2013; Osburn et al., 2019).

The carbon chemistry of ocean water is characterized by the carbon dioxide partial pressure (*p*CO_2_), total alkalinity, pH and concentrations of dissolved inorganic carbon (DIC), which includes carbon dioxide, carbonic acid, bicarbonate ions, and carbonate ions. Under increasingly acidic conditions, the concentration of carbonate ions decreases, potentially harming organisms that use carbonate to produce calcium carbonate skeletons including corals, mollusks, crustaceans, and phytoplankton. The form of calcium carbonate mineral most commonly used by organisms is aragonite or calcite; the decrease in carbonate results in a lower aragonite saturation state (Ω_arag_) or calcite saturation state (Ω_cal_) meaning that skeletons made of aragonite or calcite will be energetically more difficult to form (Kleypas et al., 2006; Fabry et al., 2008). Other studies suggest that some calcifying organisms such as mollusks and corals use other forms of DIC (e.g., bicarbonate) moreso than carbonate for calcification (Hurd et al., 2019) and that increasing proton concentrations [H^+^] (reduced pH) are more influential on calcification (Cyronak et al., 2015; Comeau et al., 2018; Hurd et al., 2019). Nonetheless, the U.S. Southeast is rich in economically important calcifying organisms that use calcium carbonate to build their shells and skeletons. Reefs formed from shellfish or corals are dominant ecosystems of the coastal region and both are particularly vulnerable to acidification (Andersson and Gledhill, 2013; Lemasson et al., 2017).

**FIGURE 1 |:**
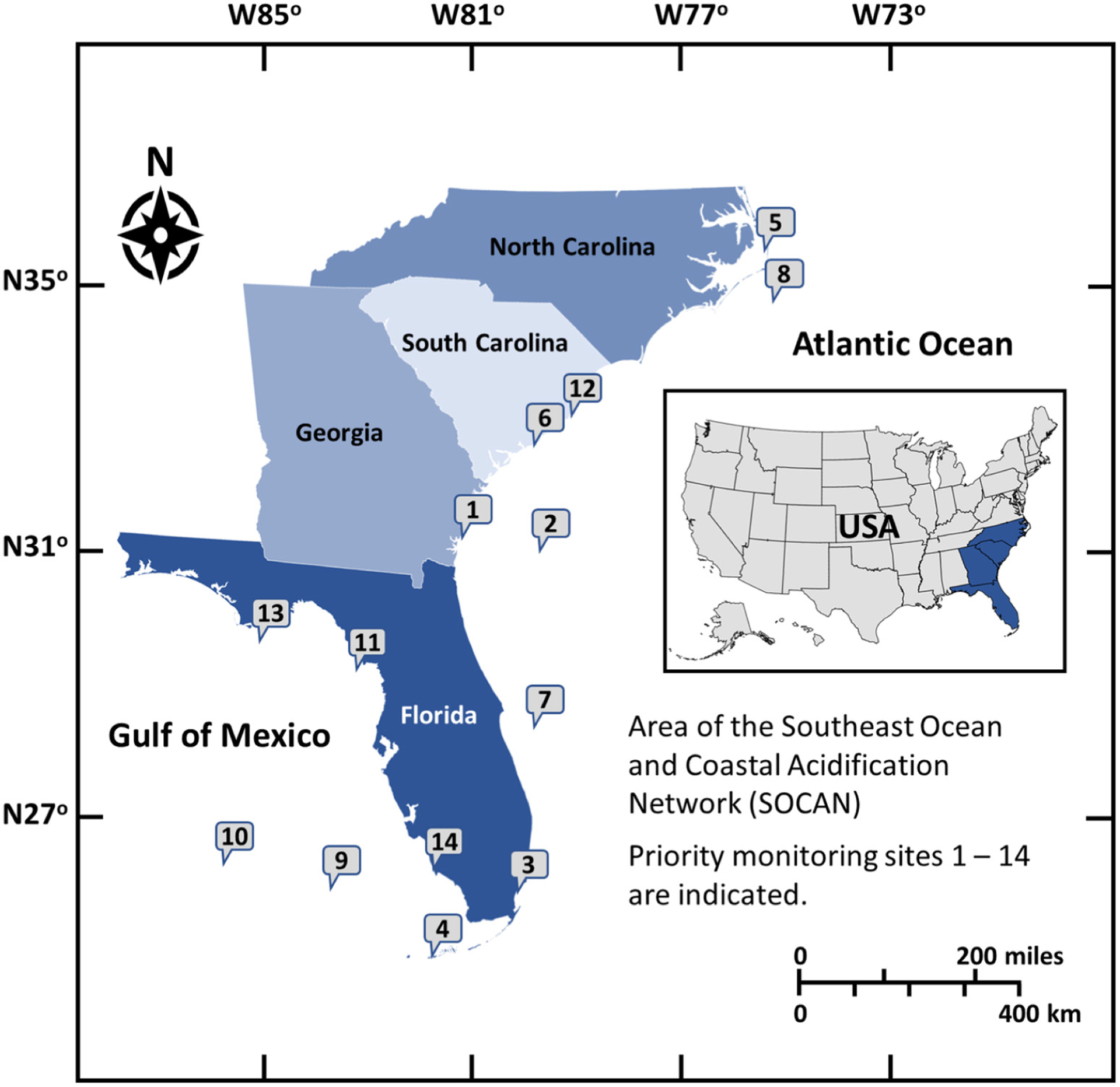
Fourteen potential monitoring sites in the Southeast listed in order of priority based on feasibility, logistics and partnerships: (1) Sapelo Island, Georgia, (2) Gulf Stream Buoy, (3) Biscayne National Park, Florida (4) Florida Lower Reef Tract, Florida, (5) Albemarle-Pamlico Sound, North Carolina, (6) Charleston Harbor, South Carolina, (7) Oculina Reefs, (8) Diamond Shoal, (9) Pulley Ridge, (10) Gulf of Mexico Loop Current, (11) Suwannee River, Florida, (12) North Inlet-Winyah Bay, South Carolina, (13) Apalachicola Bay, Florida, (14) Rookery Bay, Florida.

Regional Coastal Acidification Networks (CANs) have formed throughout the United States to build public knowledge of regional drivers and impacts of acidification and to provide a collaborative framework for stakeholders, policymakers and scientists (Cross et al., 2019). The Southeast Coastal Ocean Observing Regional Association (SECOORA) and the National Oceanic and Atmospheric Administration’s (NOAA) Ocean Acidification Program facilitated the formation of the Southeast Coastal and Ocean Acidification Network (SOCAN) to support and encourage discussions on ocean and coastal acidification in the Southeast region of the U.S. from North Carolina to Florida (Figure 1). SOCAN was formed in January 2015 and serves as both an information network and catalyst for discussions among interested parties about the state of acidification science, providing a forum for understanding and addressing coastal and ocean acidification concerns. It aims to foster enhanced communication among stakeholders within the region, including hosting a state-of-the-science webinar series and workshops on acidification. These activities have stimulated dialogue among key stakeholders to identify what is known, and what additional work is needed to further develop research and monitoring strategies. A key objective of SOCAN is to translate the global problem of acidification to regional and local scales for action. Our understanding of acidification and how to mitigate these changes at a regional scale has evolved recently to consider not only the global contribution of atmospheric carbon dioxide to the ocean but the many other mechanisms of acidification that can impact coastal waters. This document aims to describe current research on acidification in the Southeast, the current state of SOCAN, and recommendations for research and community outreach on acidification.

## ACIDIFICATION IN THE U.S. SOUTHEAST: FROM A GLOBAL PROBLEM TO A LOCAL WATER QUALITY ISSUE

### Offshore Waters

The offshore waters of the U.S. Southeast generally have lower carbon dioxide levels (260–500 μatm; Jiang et al., 2008) and higher aragonite saturation states (Ω_arag_ 2.6–4.0;Wang et al., 2013) than other U.S. regions. Compared to the U.S. West Coast, this is primarily a function of the younger water mass age of Atlantic waters, warmer temperatures, and limited upwelling (Jiang et al., 2010). The U.S. Northeast has lower aragonite saturation states as a function of lower total alkalinity waters that originate from the Labrador Sea and Canadian Shelf, while U.S. southeast regional chemistry is driven by high alkalinity waters of the Gulf Stream (Wang et al., 2013). Nevertheless, carbon dioxide levels are increasing in U.S. southeast waters, from 3.0 to 5.2 μatm yr^−1^, which is much higher than open ocean increases (Reimer et al., 2017a; Reimer et al., 2017b). Analyses of 8.5 years of data from an ocean acidification mooring at the Gray’s Reef National Marine Sanctuary suggests a rapid *p*CO_2_ increase and pH decrease, most likely related to terrestrial export into rivers, estuaries and salt marshes of both DIC and organic matter (Reimer et al., 2017b; Xue et al., 2017). Terrestrial export and enhanced microbial respiration could play a more dominant role than atmospheric CO_2_ in increasing *p*CO_2_ and decreasing pH of coastal and shelf waters in the near-future (Reimer et al., 2017a).

### Inshore Waters

Estuarine and coastal marsh waters in the Southeast region have significantly higher levels of carbon dioxide than those offshore, with surface waters near Sapelo Island, Georgia reaching 1,300 μatm (Jiang et al., 2008) and surface waters near Apalachicola Bay, Florida reaching 2,039 μatm (Joshi et al., 2018). Values become even more extreme in tidal marshes and shallow creeks such as in the Charleston Harbor estuary where summer CO_2_ levels can reach as high as 46,000 μatm (pH = 6.48) (Cochran and Burnett, 1996). These extreme conditions are primarily a function of respiratory CO_2_ from the metabolism of microbes and other marine organisms (Cochran and Burnett, 1996; Gilbert et al., 2010).

In addition to respiratory production and photosynthetic uptake of CO_2_, CO_2_ levels in water can be influenced by (1) nutrient inputs, (2) seasonal, event-based, and long-term warming, and (3) organic carbon export from land. In oceanic waters on the middle shelf and possibly in the Gulf Stream, warming likely contributes to an increase in *p*CO_2_ and decrease in pH (Reimer et al., 2017a). Warming increases microbial oxidation rates and this enhanced breakdown of organic matter increases the *p*CO_2_ in coastal waters. Export of CO_2_-rich water from estuaries is a key element identified by Reimer et al. (2017a) contributing to acidification in coastal waters. It is likely that warming will continue to enhance respiration rates and their contribution to coastal acidification. The potential exists for sea level rise to result in enhanced “liberation” of organic material and respiratory CO_2_, further compounding acidification trends in coastal waters (Reimer et al., 2017a).

In North Carolina, nutrient enrichment has caused enhanced primary production at the surface, and eventually acidification trends in bottom water (Van Dam et al., 2019). Eutrophication-based acidification is an important contributing factor to consider as rapidly increasing human populations are expected to lead to increasing eutrophication. Historically, only four southeast estuarine systems are characterized as highly eutrophic, but more than half of the estuaries currently exhibiting low to moderate eutrophic conditions are expected to worsen (Bricker et al., 2008). However, studies show eutrophication-based acidification may be limited to stratified estuaries (Feely et al., 2010; Cai et al., 2011; Van Dam and Wang, 2019) which could be important because eutrophication can potentially offset acidification in well-mixed systems (Nixon et al., 2015).

The carbonate chemistry of waters in the Southeast is strongly affected by the total alkalinity of river water that discharges at the coast. During rain events, in regions where land has been developed, water that would otherwise be absorbed into the ground flows instead into surrounding streams, rivers, and estuaries causing dramatic and more extreme changes in temperature, salinity, dissolved oxygen, and pH. Increasing freshwater flow can lead to reduced carbonate ion concentrations and reduced buffering capacity in coastal waters (Salisbury et al., 2008). The southeast is expected to see moderate increases in precipitation in coastal regions and an increase in heavy precipitation events within the next century (USGRP, 2018). This region has the largest rate of population change of any U.S. region (U. S. Census Bureau, 2015), leading to an increase in impervious surfaces and changes to the hydrological cycle (Holland et al., 2004). But longer-term trends in increased river drainage are not always associated with acidification trends. In two neighboring North Carolina river systems, Van Dam and Wang (2019) found increased river discharge coincided with an acidification trend in the Neuse River estuary but not the neighboring New River estuary. Increasing total alkalinity (“buffering capacity”) relative to DIC in the New River estuary likely buffered against carbon dioxide increases (Van Dam and Wang, 2019).

Tropical cyclones have had increasingly heavier precipitation associated with them, with impacts inland, extended flooding and heavy contamination (Paerl et al., 2019). Terrestrial organic matter and its degradation to CO_2_ persisted in the Neuse River Estuary-Pamlico Sound for several months after Hurricane Matthew in 2016 (Osburn et al., 2019). Paerl et al. (2018) found that during storm years, this estuary is a significant source of CO_2_ to the atmosphere while in storm-free years it is a sink. In the 14- day flood period from Hurricane Joaquin, CO_2_ emissions were 31 and 44% of the total annual CO_2_ flux for the Neuse River Estuary and New River Estuary, respectively (Van Dam et al., 2018). Furthermore, major storms double nitrogen- and triple phosphorus-loading to the estuary compared to storm-free years (Paerl et al., 2018).

The carbonate chemistry of shallow coastal waters in Florida is strongly influenced by seagrasses, coral reefs, carbonate sediments, and the carbonate platform upon which the Florida peninsula sits. In Florida estuaries associated with shellfish harvesting, Robbins and Lisle (2018) found that pH decreased in 8 out of 10 estuaries from 1980 to 2008 but rates of decrease were 2.0–3.4 times slower than offshore waters, possibly mitigated by the carbonate platform’s contributions to buffering capacity. In Tampa Bay, the concentration of carbonates in sediments is lower but seagrass beds locally elevate pH and carbonate saturation states compared to bare sand habitats (Yates et al., 2016). Despite this potential buffering effect, measurements at Saint Joseph Bay have shown *p*CO_2_ can reach as high as 2,537 μatm and pH as low as 7.36 with diel variability ranging from 379 to 1,019 Ωatm (Challener et al., 2016). Van Dam et al. (2019) found that some seagrass beds in Florida Bay are in fact net heterotrophic, indicating that carbonate dissolution and respiration in sediments exceeds the net productivity of seagrasses and calcification. Inshore Florida Reef Tract (FRT) sites showed net uptake of total CO_2_ from photosynthesis in the spring and summer with concomitant increases in Ω_arag_ despite reduced total alkalinity (Manzello et al., 2012).

## THE VULNERABLE ORGANISMS AND ECOSYSTEMS OF THE U.S. SOUTHEAST

The Southeast has economically important organisms that are especially vulnerable to acidification, including shellfish, corals, and crustaceans. There have been recent efforts to grow the shellfish industry in the southeastern U.S. (e.g., Baillie et al., 2018). Corals in the FRT provide an asset value of $7.6 billion (Johns et al., 2001) and $1.6 billion coastal hazard protection from severe storms (Storlazzi et al., 2019). Crustaceans such as stone crabs can contribute up to ~$30 million a year to Florida’s economy alone (Gravinese et al., 2018). Other organisms in the Southeast that may also be impacted by acidification include. Mollusks, finfish and elasmobranchs (Saba et al., 2019) and possibly microbes (Liu et al., 2010; Krause et al., 2012) and plankton, including harmful algal species such as *Karenia brevis* (Florida Red Tide; Errera et al., 2014).

Larval hard clams (*Mercenaria mercenaria*) are negatively influenced by acidification showing dramatic declines in survivorship, delayed metamorphosis, and size reduction, even at relatively moderate levels of CO_2_ (650 μatm) but more so at higher levels (1,500 μatm) (Talmage and Gobler, 2009) that commonly occur in southeastern estuaries. Mechanical properties of juvenile clams’ shells (microhardness and fracture toughness) were negatively affected when exposed to elevated CO_2_ (800–1,500 μatm) in laboratory experiments (Dickinson et al., 2013). There may be tipping points as demonstrated by laboratory experiments with the eastern oyster, *Crassostrea virginica*, that show acidification impacts on survival (Talmage and Gobler, 2009) and on processes that are metabolic and mineralogical (Beniash et al., 2010), and proteomic (Tomanek et al., 2011). Oyster reproduction appeared to be resilient to open ocean projections of pH change, but “severe” treatments (pH 7.1) showed significant negative effects on reproduction (Boulais et al., 2017). Such extreme pH/CO_2_ conditions are common in coastal marsh habitats in the summer.

Another commercially-important inhabitant is the Atlantic blue crab, *Callinectes sapidus*. When the ambient *p*CO_2_ was raised from 800 to 8,000 μatm, juvenile blue crabs showed no sensitivity to the duration of the intermolt period or to food consumption rate (Glandon and Miller, 2017). Additional studies using the same *p*CO_2_ changes showed small but significant changes in the high-magnesium calcite weight in the carapace of juvenile blue crabs and an increase in magnesium content (Glandon et al., 2018). In adult blue crabs, performance was judged by quantifying fatigue behaviors using underwater treadmills and by quantifying mate-guarding behavior (Stover et al., 2013). Performance declined in moderate hypoxia (50% air saturation), however, when *p*CO_2_ was elevated to 19,700 μatm in the same hypoxic conditions, performance improved. Lehtonen and Burnett (2016) suggested that improvement was due to a CO_2_-specific effect on the hemocyanin that increases oxygen affinity. Other studies show a reduction in size and survival of blue crab larvae (Glitz and Taylor, 2017) and thus a sensitivity to acidification. Stone crabs (*Menippe mercenaria*) are also vulnerable to acidification, especially at different life-stages, slowing down embryonic development, reducing hatching success, and decreasing larval survivorship (Gravinese, 2018; Gravinese et al., 2018).

Florida is the only state in the continental U.S. to have extensive shallow coral reef formations near its coasts (Florida Department of Envrionmental Protection, 2011). Coral reefs have been among the most well-studied ecosystems with regards to impacts from ocean acidification (reviewed by Kleypas and Yates, 2009; Hoegh-Guldberg et al., 2017). Coral calcification rates can be negatively impacted with declines of 20–30% per unit change in Ω_arag_ in the laboratory (Langdon and Atkinson, 2005; Kroeker et al., 2010) and perhaps moreso with decreases in seawater pH and associated problems of pH homeostasis within organisms under acidification conditions (Cyronak et al., 2015; Comeau et al., 2018), but the results may not directly translate to changes in community net calcification. While most of the FRT shows net carbonate production, reef calcification is just 10% of its historical rate and the northernmost reefs are subject to net dissolution (Muehllehner et al., 2016). Regional scale measurements of seafloor elevation change throughout the FRT show significant erosion of coral reefs and the surrounding seafloor that (combined with sea level rise) has caused water depths to increase to levels not expected until near the year 2100, indicating that carbonate production is insufficient to keep pace with erosion and sea level rise (Yates et al., 2017). Comparisons of vertical reef growth potential to sea level rise projections indicate that significant reef submergence (increased water depth) will occur by 2100 under Representative Concentration Pathway (RCP) 4.5 and 8.5 scenarios (Perry et al., 2018); and the coastline protection that reefs provide through wave energy attenuation could be greatly reduced with an increase in water depth over the reef (Ferrario et al., 2014). Wound recovery, reproduction, and recruitment in corals are also impacted by acidification and will negatively affect reefs of the FRT (Albright et al., 2010; Enochs et al., 2014; Hall et al., 2015).

The U.S. Southeast harbors the most expansive cold-water framework-forming reefs in the United States (Hain and Corcoran, 2004; Ross and Nizinski, 2007), and preliminary comparisons between coral locations (NOAA DSCRTP Deep Sea Coral Data Portal)^1^ and hydrographic cruise data (Wanninkhof et al., 2015) provide evidence that low Ω_arag_ waters are impinging on these habitats. *Oculina* Banks (*Oculina varicosa*) are found along the shelf edge off the east coast of central Florida between 70 and 100 m (Reed, 2002b). These diverse reefs form important nursery grounds and habitat for commercially-important fish and were designated as a Habitat Area of Particular Concern in 1984 with a large expansion in 1994. In deeper waters along the coast from Florida to South Carolina, *Lophelia pertusa* and *Enallopsammia profunda* reefs are found between 490 and 870 m (Reed, 2002a). Studies in the Gulf of Mexico found intra-specific variation of *L. pertusa* calcification due to low pH in laboratory experiments (Lunden et al., 2014; Kurman et al., 2017) and survival under *in situ* aragonite saturation of 1.3 (Georgian et al., 2016), but these studies do not account for the potential loss of framework from dissolution (Hennige et al., 2015). To date there have been no published studies evaluating carbonate chemistry dynamics and effects of ocean acidification on cold-water corals in the South Atlantic Bight.

Uncertainties remain on how ocean acidification may affect plankton, including harmful algal species, and microbial communities. Bacterial assemblages, phyto- and zooplankton might be affected directly (e.g., physiological changes) or indirectly (e.g., changes in food availability, community structure, size classes and stoichiometry) (Feng et al., 2009; Hutchins et al., 2009; Caron and Hutchins, 2012). Suggestions have been made that elevated *p*CO_2_ could shift plankton assemblages to more harmful species such as Florida red tide, which can cause human respiratory distress, fish and other marine-life kills, and disrupt coastal economies (Weisberg et al., 2019). Laboratory studies have shown increases in growth rates of Florida red tide (*Karenia brevis*) with elevated *p*CO_2_ (Errera et al., 2014). Multiple stressor studies confirm that changes in pCO_2_ alone do not render the same responses in single plankton or microbe species or complex assemblages as do conditions where temperature and/or nutrient levels are also manipulated (e.g., Riebesell et al., 2008; Tatters et al., 2013). Moreover, it becomes apparent that multiple stressors affect coexisting species differently, making it difficult to predict the overall outcome on plankton community structure and functionality on each trophic level (Tatters et al., 2013; Andersson et al., 2015) and to definitively attribute changes to ocean acidification (Doo et al., 2020). Combining experimental approaches with molecular and modeling tools has begun to provide some insight into how plankton and microbes can adapt to changes in pH (Andersson et al., 2015; Dutkiewicz et al., 2015).

## LOW DISSOLVED OXYGEN AND ACIDIFICATION

Hypoxia can often accompany elevated *p*CO_2_ in coastal waters primarily resulting from oxygen consumption by respiration at night when photosynthesis declines and CO_2_ fixation decreases (e.g., Cochran and Burnett, 1996; Burnett, 1997; Cai et al., 2011; Sunda and Cai, 2012; Melzner et al., 2013). Hypoxia can also occur on seasonal timescales due to eutrophication and decay processes (Gray et al., 2002). Some of the experimental results cited above suggest negative impacts of low dissolved oxygen on the development, performance, and survival of larval forms and adult organisms. Hypoxia is a large and growing problem in coastal waters throughout the world (Diaz and Rosenberg, 2008) and in combination with elevated *p*CO_2_ it can be especially challenging to organisms. Since *p*CO_2_- driven acidification frequently co-occurs with hypoxia, as a consequence of coastal eutrophication and intense respiration in bottom waters (Sunda and Cai, 2012; Duarte et al., 2013), these findings suggest yet another threat to estuarine organisms. Here we highlight examples of this consideration within the Southeast region.

Elevated *p*CO_2_ with low oxygen can change the toxicity of some pesticides. Garcia et al. (2014) examined the toxicity of several mosquito control agents on larval and juvenile clams and oysters and found that hypoxia and increased *p*CO_2_ levels alone or in combination caused mortality in larval clams and increased the toxicity of resmethrin, a mosquito-control pyrethroid insecticide. These results underscore the confounding role ocean acidification may play in future climate change scenarios in the dynamically changing landscape ecology of the Southeast U.S. coastal zone.

New evidence from studies of gene expression suggests that elevated *p*CO_2_ can have deleterious effects on the adaptive responses of some organisms to hypoxia. An adaptive response of organisms with a respiratory pigment (e.g., hemoglobin, hemocyanin) is to produce more pigment in hypoxia. Studies have shown that this adaptive response to hypoxia is muted with elevated *p*CO_2_ (Rathburn et al., 2013; Johnson et al., 2015).

## SOCIOECONOMIC IMPACTS IN THE SOUTHEAST

Though the extent of their vulnerability in the U.S. Southeast is yet to be determined, shrimps, crabs and mollusks represent a large majority of the commercially-harvested species in the region. As marine calcifiers, each of these marine invertebrate groups have been recognized as potentially vulnerable to acidification (Guinotte and Fabry, 2008). They represent not only an economic resource, but also a culturally important resource in the region. Despite the diversity of marine organisms in the Southeast, the low diversity of harvested marine species could render coastal communities vulnerable. Furthermore, shellfish provide a multitude of ecosystem services, including water filtration, pathogen and pollution removal, shoreline stabilization, storm protection, and are an iconic indicator of environmental health (Scott and Lawrence, 1982; Cooley and Doney, 2009; Lemasson et al., 2017).

Research that evaluated vulnerability and adaptive capacity of U.S. shellfisheries to ocean and coastal acidification ranked North and South Carolina as relatively high risk compared to other coastal U.S. states (Ekstrom et al., 2015). According to these authors, the Carolinas ranked high due to at least ten estuaries with a high eutrophic score and/or low carbonate (aragonite saturation) river drainage that can locally amplify acidification. The Southeast ranked medium to high in sensitivity for economic dependence on shellfish and among the lowest for adaptive capacity. Combined, these rankings indicate a high social vulnerability to acidification in North Carolina with decreasing rankings moving geographically south, due largely to a decreased dependence on shellfish (Ekstrom et al., 2015). Florida was assigned a relatively low social vulnerability score because of low economic dependency on shellfish and ample alternative employment. However, Florida coral reefs represent an economically-important resource and have already experienced carbonate dissolution on a seasonal level (Muehllehner et al., 2016).

The oyster industry currently generates $7.5 million in economic activity in the U.S. Southeast (Van Voorhees et al., 2015) but has the potential to expand to over $100 million by 2030 just in North Carolina alone (Baillie et al., 2018). North Carolina’s Strategic Plan for Shellfish Mariculture cites maintaining and improving water quality as a key recommendation, noting that in 2017, 19% of growing areas were closed due to poor water quality and 14% closed because of lack of funding to monitor water quality (Baillie et al., 2018). Though not explicitly noted in the report, many of the measures to protect water quality (i.e., stormwater system maintenance, low impact development) would also mitigate acidification.

The economic costs of acidification have been well-quantified for coral reef ecosystems. The Florida Keys reefs are estimated as having a $7.6 billion asset value supporting approximately 71,000 jobs (Johns et al., 2001). The ecosystem services these reefs provide are dependent on their three-dimensional structure. Degradation of these structures has been attributed to both local stressors; such as pollution, overfishing, land run-off, physical damage and disease, as well as global stressors, including sea level rise, acidification and warming ocean temperatures. Predicted economic losses based on surveys in the Florida Keys from climate change and acidification impacts include household safety concerns, tourism loss, property loss, abandonment, and flooding impacts (Mozumder et al., 2011). Reef loss will also disrupt ecosystem regulating services such as carbon sequestration, food web regulation, and coastal protection (e.g., Rodrigues et al., 2013). Few studies have focused on socioeconomic impacts from acidification and those that do have mainly focused on fisheries impacts (Rodrigues et al., 2013). Acidification may also affect public health and security with increased shifts of marine parasites that can reduce food sources from coral reefs (Gattuso et al., 2015).

## SOCAN: A REGIONAL NETWORK TO SUPPORT THE SOUTHEAST ACIDIFICATION COMMUNITY

### Establishing SOCAN

SOCAN was established in 2015 with support from SECOORA and NOAA’s Ocean Acidification Program. Since its foundation, SOCAN has synthesized Southeast acidification research, established regional acidification monitoring and research priorities, and developed partnerships with regional stakeholders. Given the relative dearth of acidification impact research in the U.S. Southeast, SOCAN has recently focused on facilitating the development of collaborative research and monitoring activities to help understand and manage the biological and societal impacts specific to this region.

After initial organization of the network in 2015, which included hosting a total of 18 state-of-the-science ocean acidification webinars on the SOCAN website^2^, SOCAN held its first in-person meeting in January 2016 to evaluate the state of the science and establish research priorities. The top acidification research priorities identified by SOCAN included:

1. Monitoring key ocean parameters across various spatial and temporal scales that will provide information on mechanistic drivers of acidification (i.e., river discharge, eutrophication, upwelling, atmospheric uptake of CO_2_, biological consumption/production), and input parameters for predictive model algorithm development.
2. Establishing a robust experimental approach for organismal response that includes a multi-stressor design and an understanding of acclimation vs. adaptation with attention to scaling. By identifying standard operating procedures, SOCAN can unify experimental approaches to better cross compare species.
3. Developing biogeochemical, operational and qualitative models that can transition to end users and adapting existing models to understand acidification.

As evidenced in the previous sections, there have been significant advancements in understanding mechanistic drivers of acidification. Progress has been made since 2015 in identifying sources of variability from a regional perspective (Reimer et al., 2017a), as well as in specific systems (Challener et al., 2016; Reimer et al., 2017a,b; Yates et al., 2017; Van Dam et al., 2018, 2019; Van Dam and Wang, 2019). There is much work to be done to link these changes to biological impacts and to understand subsequent consequences for human communities.

### Advancing Monitoring Priorities

SOCAN partners established priorities for key monitoring locations that would provide critical data for acidification science and information for stakeholders (Wickes, 2017a). Fourteen potential monitoring sites were selected throughout the region (Figure 1). These locations represent key end members or transition zones along climate and environmental gradients that affect, and are affected by, acidification. The three highest priority sites based on feasibility, logistics and partnerships are briefly described below:

Priority 1 – The first proposed site is the coastal zone of Sapelo Island, Georgia, which has proximity to the existing mooring at Gray’s Reef National Marine Sanctuary (GRNMS). The paired continuous monitoring locations would provide key insights into causal factors of changing chemistry (e.g., location of Gulf Stream vs. river drainage) and the region has important shellfish farms that have partnered with SOCAN to help inform monitoring and research activities.

Priority 2 – The second proposed site includes either a new mooring farther offshore of GRNMS or an upgraded mooring offshore of Edisto Island, SC. These locations would allow for measurement of chemical changes in the Gulf Stream, a dominant and important regional feature in determining the regional chemistry.

Priority 3 – The third proposed site is Biscayne National Park, at the northern edge of the Florida Reef Tract coinciding with locations that showed seasonal net dissolution of the reef and highest rates of reef and seafloor erosion (Muehllehner et al., 2016; Yates et al., 2017).

### SOCAN Stakeholders

SOCAN workshops have stimulated significant discussion of the heterogeneity of acidification in the coastal region and its impact on translating available scientific information to an actionable response for stakeholders. Diffuse and inland sources of nutrients and other pollutants create an inherent problem in organizing management of coastal water quality. However, relative to regulations of global anthropogenic CO_2_, land- based sources of acidification present an opportunity for state and local-level mitigation. SOCAN worked with state partners to identify those programs that monitor and regulate water quality and have indirect linkages to potential acidification, such as state programs that regulate nutrient loads and monitor hypoxia. Existing water quality monitoring programs provide an opportunity to leverage additional efforts to monitor carbonate chemistry. Though the workshops were clear evidence of the diversity of stakeholders interested in acidification issues in the U.S. Southeast, most stakeholders suggested there is not yet enough information on localized carbonate chemistry dynamics and species responses within the Southeast region to guide an actionable response.

According to North Carolina stakeholders, there are accounts of sudden and significant losses of shellfish at mariculture farms that have not been accounted for by disease or other commonly measured water parameters such as temperature and salinity (Wickes, 2017b). These events are typically not reported in a timely manner to a centralized database and the lack of water quality measurements at shellfish farms limits identification of causal agents of mortality (Barton et al., 2015). SOCAN is seeking to leverage partnerships with local universities and non-profits to work toward including carbonate chemistry with parameters already measured at shellfish farms to monitor for potential impacts from acidification.

### SOCAN Looking Forward

While information on regional and estuarine carbonate chemistry has quickly evolved, there is a scarcity of data on potential biological and socioeconomic impacts of acidification in this region. SOCAN is currently pursuing partnerships and funding opportunities to advance biological research in association with shellfish hatcheries and industry partners. In addition to a dearth of information on the response of U.S. Southeast shellfish to acidification, there are also significant gaps in our understanding of acidification impacts on harmful algal blooms, finfish, and microalgae in the region as well as identifying areas of refugia and community level responses. Acidification must be considered in combination with warming, high precipitation events, sea level rise, eutrophication, and hypoxia as investigations of single drivers can sometimes produce misleading results. Though many studies have shown impacts of acidification in the laboratory, there has been very little research conducted on Southeast stocks of shellfish and no *in situ* studies to connect changing chemistry with biological impacts to shellfish in the region.

A long-term aim of SOCAN is to expand efforts to develop models from existing data that can be used to determine the biological, chemical, and physical drivers of acidification. Identifying the relationships between coastal acidification conditions and other water quality parameters will provide a foundation for how acidification could be considered within existing water quality regulations. Due to the current relative importance of coastal acidification mechanisms (i.e., eutrophication, freshwater input) over atmospheric inputs, the Southeast has a variety of tools to address these localized contributions to changing coastal chemistry. Existing regulations for point and non-point source pollution, wastewater treatment and initiatives to address impervious surfaces can contribute to curbing acidification. As the research evolves, SOCAN has a role in translating coastal acidification, chemical and biological impact data to a holistic understanding of potential consequences for coastal communities. Working with both scientific and natural resource management partners SOCAN can translate scientific models to operational models.

In closing, SOCAN offers the following recommended emphasis on current acidification research priority foci specific to the Southeast region:

Multiple and/or compounding drivers of acidification;
Single organism and community investigation on impacts of acidification;
Life-stage analyses of “important” organisms;
Effects of acidification on harmful algae species;
Buffering capacity of coastal areas to acidification;
Monitoring of estuarine and riverine acidification;
Social and economic impacts from acidification; and
Partnering with local management and industry to understand local impacts of acidification.

## Footnotes

1 https://deepseacoraldata.noaa.gov/

2 https://www.socan.secoora.org/
